# Sex difference in the association between metabolic syndrome and the risk of stroke: a systematic review and meta-analysis

**DOI:** 10.3389/fneur.2025.1527749

**Published:** 2025-06-04

**Authors:** Lingling Zhang, Qi Chi

**Affiliations:** ^1^Department of Neurology, South China Hospital, Medical School, Shenzhen University, Shenzhen, China; ^2^Department of Radiology, South China Hospital, Medical School, Shenzhen University, Shenzhen, China

**Keywords:** sex difference, metabolic syndrome, stroke, systematic review, meta-analysis

## Abstract

**Background:**

Metabolic syndrome comprises multiple cardiovascular risk factors, and previous studies have confirmed a significant association between metabolic syndrome and an increased risk of stroke. However, no systematic meta-analysis has evaluated the sex differences in the relationship between metabolic syndrome and stroke. This study aimed to investigate the sex difference in the association between metabolic syndrome and stroke.

**Methods:**

The PubMed, Embase, and Cochrane Library databases were systematically searched for eligible studies until October 2024. The sex difference in the association between metabolic syndrome and the risk of stroke was calculated by relative risk ratio (RRR) with 95% confidence interval (CI) using a random-effects model with inverse variance weighting.

**Results:**

Nine studies involving 61,060 individuals were included in the meta-analysis. No sex difference was observed in the association between metabolic syndrome and the risk of stroke (RRR: 0.92; 95% CI: 0.72–1.17; *p* = 0.482). Sensitivity analysis found that the sex difference in this association was stable. Subgroup analyses revealed that male individuals with metabolic syndrome had a greater risk of stroke than female individuals in studies with a follow-up duration of <10.0 years (RRR: 0.70; 95% CI: 0.46–1.04; *p* = 0.078) and with low quality (RRR: 0.54; 95% CI: 0.30–0.98; *p* = 0.043).

**Conclusion:**

Sex differences may exist in the association between metabolic syndrome and the risk of stroke, especially with shorter follow-up periods. Further large prospective studies should be performed to verify the sex difference in the association between metabolic syndrome and the risk of stroke.

## Introduction

Stroke remains the second leading cause of death globally, with approximately 6.55 million deaths attributed to stroke (1). In China, nearly 17.8 million people experience stroke, resulting in 2.3 million deaths (2). The prevalence of stroke in the general population is 2.6%, but this figure surges to 44.8% among individuals with hypertension (3). Ischemic stroke and transient ischemic attacks account for nearly 87% of all stroke cases (4). The major complications following a stroke include motor dysfunction, sensory impairment, and dysphagia (5). According to estimates, globally, there are 4.5 million deaths due to stroke each year and more than 9 million stroke survivors (6). Therefore, exploring the risk factors for stroke to implement effective primary prevention is crucial.

Metabolic syndrome (MetS) is a significant risk factor for vascular diseases, including stroke (7). The primary characteristics of MetS include central obesity, elevated triglyceride levels, diabetes or insulin resistance, hypertension, and reduced high-density lipoprotein cholesterol (HDL) levels (8). Research indicates that age, sex, smoking habits, alcohol consumption, and physical activity frequency are factors associated with the development of MetS (9). As global concern over MetS as a public health challenge continues to grow, its prevalence is estimated to range from 14 to 30% (10). Specifically, in Iran, data from 2020 show that the prevalence of MetS reached 30.8% (11). Studies suggest that the inflammatory processes observed in patients with MetS may contribute to the progression of cardiovascular diseases, partly owing to dysregulated fat metabolism (10). Previous studies have confirmed that MetS significantly increases the risk of stroke, but whether this association differs by sex remains unknown (12).

Notably, potential sex differences in stroke risk may be attributed to several underlying biological mechanisms. Hormonal factors play a crucial role. In pre-menopausal women, higher estrogen levels offer protection to the cardiovascular system. Estrogen can regulate vascular dilation, inhibit inflammation, and reduce oxidative stress, thereby lowering the risk of stroke. However, after menopause, the decline in estrogen levels weakens this protective effect, leading to an increased risk of stroke (13). In terms of genetic factors, although research is currently limited, some genetic polymorphisms may potentially affect stroke risk differently in men and women, yet the specific mechanisms remain to be further explored. Environmental factors also cannot be overlooked. Lifestyle differences, such as smoking, excessive alcohol consumption, and physical inactivity, which are more prevalent in men in some cases, and social-psychological stress, which may have a greater impact on women, can contribute to the observed sex differences in stroke risk (14). Therefore, we conducted this systematic review and meta-analysis to assess the sex difference in the association between MetS and the risk of stroke.

## Materials and methods

### Search strategy and selection criteria

This study adhered to the Preferred Reporting Items for Systematic Reviews and Meta-Analysis guideline (15). This study investigated the sex difference in the association between MetS and the risk of stroke and reported this relationship in males and females. Moreover, the publication language and status were not restricted. We systematically searched PubMed, Embase, and the Cochrane Library to identify relevant studies published up to October 2024 using the following search terms: (“cardiovascular risk” OR “cardiovascular disease” OR “stroke”) AND (“metabolic syndrome”) AND (“cohort studies” OR “prospective studies”). Reference lists of the included studies were also manually reviewed for additional studies that met the inclusion criteria.

Two reviewers independently performed the literature search and study selection, and any disagreement was settled through mutual discussion. A study was included if it met the following criteria: (1) patients: free of stroke at baseline; (2) exposure: MetS; (3) control: non-MetS; (4) outcome: stroke incidence or effect estimate in the relationship between MetS and the risk of stroke; (5) study design: prospective cohort; and (6) additional criteria: reported the relationship between MetS and the risk of stroke in males and females simultaneously. Review, letter, and animal experiment studies were removed owing to irrelevant or no relevant data. It is important to note that for studies that did not report sex-specific outcomes, we did not contact the authors for additional data. These studies were excluded from the analysis to ensure that our meta-analysis focused on the sex difference in the association between MetS and the risk of stroke. In addition, it should be noted that most of the original studies included in this review did not clearly define whether their population was based on biological sex or reported gender. Considering the biological mechanisms we are investigating, we assume that the populations in these studies were defined by biological sex.

### Data collection and quality assessment

The data were independently collected by two reviewers, and they included the first author or study group’s name, publication year, country, sample size, mean age, male, smoking, body mass index, hypertension, diabetes mellitus, reported outcomes, number of stroke, adjusted factors, and follow-up duration. To assess the methodological quality of each study, we used the Newcastle-Ottawa Scale (NOS). The NOS evaluates studies based on three aspects: selection (with 4 items), comparability (1 item), and outcome (3 items) (16). Two reviewers independently carried out the quality assessment. In case of any disagreements between the reviewers regarding the data collected and quality assessment, an additional reviewer resolved the issues by referring to the original article.

### Statistical analysis

The sex-specific effect estimate with the 95% confidence interval (CI) in published studies was assigned to assess the sex difference in the association between MetS and the risk of stroke. The male-to-female relative risk ratio (RRR) was then calculated using the random-effects model, taking into account that the underlying factors vary across included studies (17, 18). The heterogeneity of the included studies was assessed using the *I^2^* and Q statistics, and significant heterogeneity was denoted by *I^2^* > 50.0% or *p* < 0.10 (19, 20). The stability of the pooled conclusion was assessed by a sensitivity analysis through sequential removing single study (21). For each removal, the RRR and 95% CI were recalculated to evaluate whether the overall result was sensitive to the exclusion of any particular study. Subgroup analyses were performed based on the country, mean age, smoking proportion, adjusted level, reported outcomes, follow-up duration, and study quality. The division by country aimed to account for differences in regional factors such as healthcare systems, lifestyle, and genetic backgrounds. Mean age was considered because the impact of MetS on stroke risk may change with age. Smoking proportion and adjusted level are known confounders, and their inclusion helps to better understand the relationship between MetS and stroke risk. Different reported outcomes (total stroke vs. ischemic stroke) were analyzed separately due to their potentially distinct underlying mechanisms. Follow-up duration was a key factor as the association between MetS and stroke risk may vary over time. Study quality was included to assess the influence of the reliability of the studies on the results. Publication biases were evaluated using the funnel plots, Egger, and Begg tests (22, 23). All the reported *p*-values are two-sided, and the inspection level for the pooled results was 0.05. All the statistical analyses in this study were performed using STATA (version 12.0; Stata Corporation, College Station, TX, USA).

## Results

### Literature search and study selection

**Figure 1:**
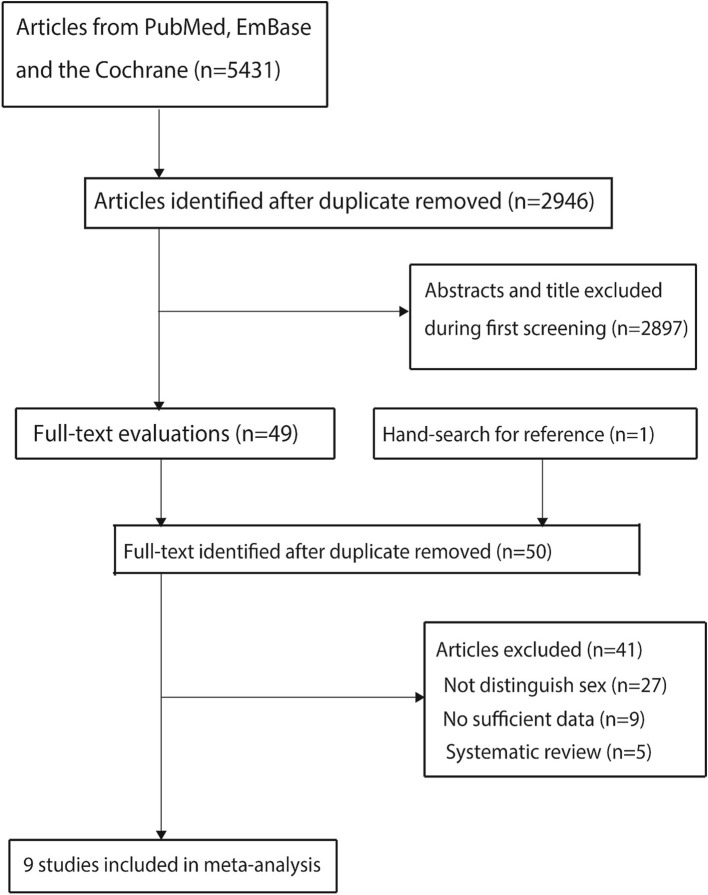
Literature search and study selection process.

The initial search yielded 5,431 articles, of which 2,946 were retained after duplicate records were removed. Subsequently, 2,897 studies were excluded owing to irrelevant titles or abstracts. The remaining 49 studies were downloaded for further full-text evaluations, and 1 study was discovered by reviewing the reference lists; 41 studies were excluded. The remaining 9 studies were used for the final meta-analysis (24–32). The details of the literature search and study selection are shown in Figure 1.

### Study characteristics

The characteristics of the included studies and involved patients are summarized in Table 1. The included studies involved a total of 61,060 individuals and 1,770 cases of stroke. The sample sizes of the included studies ranged from 1,424 to 19,369, and the follow-up duration ranged from 3.4 to 18.0 years. Six studies reported the total stroke, while 5 studies reported ischemic stroke. Two studies were conducted in the US, 3 in Japan, 2 in China, 1 in Korea, and 1 in Iran. Study quality was assessed using the NOS; 2 studies had 8 stars, 5 had 7 stars, and the remaining 2 had 6 stars.

**Table 1:** The baseline characteristics of identified studies and involved patients.

Study	Country	Sample size	Age (years)	Male (%)	Smoking (%)	BMI (kg/m^2^)	Hypertension (%)	DM (%)	Reported outcomes	No of stroke	Adjusted factors	Follow-up (years)	Study quality
ARIC 2005 (24)	US	12,089	54.0	43.1	25.6	27.7	38.9	11.0	Total stroke, IS	308/216	Age, race/center, LDL and smoking	11.0	8
JDCS 2005 (25)	Japan	1,424	58.4	54.1	27.7	22.7	25.2	100.0	Total stroke	59	Age	8.0	7
Iso 2007 (26)	Japan	9,087	51.6	39.6	27.2	23.2	11.4	NA	IS	256	Age, sex, community, serum TC, smoking, alcohol intake, time since last meal and for women, menopausal status.	18.0	7
Hisayama 2007 (27)	Japan	2,452	58.3	42.8	25.4	22.9	40.1	9.1	Total stroke, IS	209	Age, proteinuria, electrocardiogram abnormalities, TC, smoking, alcohol intake, and regular exercise	14.0	8
NOMAS 2008 (28)	US	3,297	69.0	37.0	15.0	NA	NA	17.0	IS	176	Age, education, insurance status, any physical activity, smoking, moderate alcohol use, and cardiac disease	6.4	7
SPRIS 2011 (29)	China	19,369	52.9	52.8	21.2	25.2	43.9	NA	Total stroke	341	Age, serum TC, current smoking, regular alcohol drinking, AF, family history of CVD	3.0	7
SMSRI 2012 (30)	Korea	6,430	48.9	57.9	31.4	23.5	NA	NA	Total stroke	188	Age, smoking status, alcohol drinking, exercise, and HOMA-IR	10.0	6
Yao 2016 (31)	China	1,514	57.7	52.6	22.1	24.0	NA	100.0	Total stroke	142	Age	10.0	6
ICS 2017 (32)	Iran	5,398	50.7	48.7	16.2	26.7	29.1	9.8	IS	91	Age, diet, physical activity, and smoking	10.0	7

AF, atrial fibrillation; BMI, body mass index; CVD, cardiovascular disease; DM, diabetes mellitus; LDL, low density lipoprotein; TC, total cholesterol.

### Meta-analysis and sensitivity analysis

**Figure 2:**
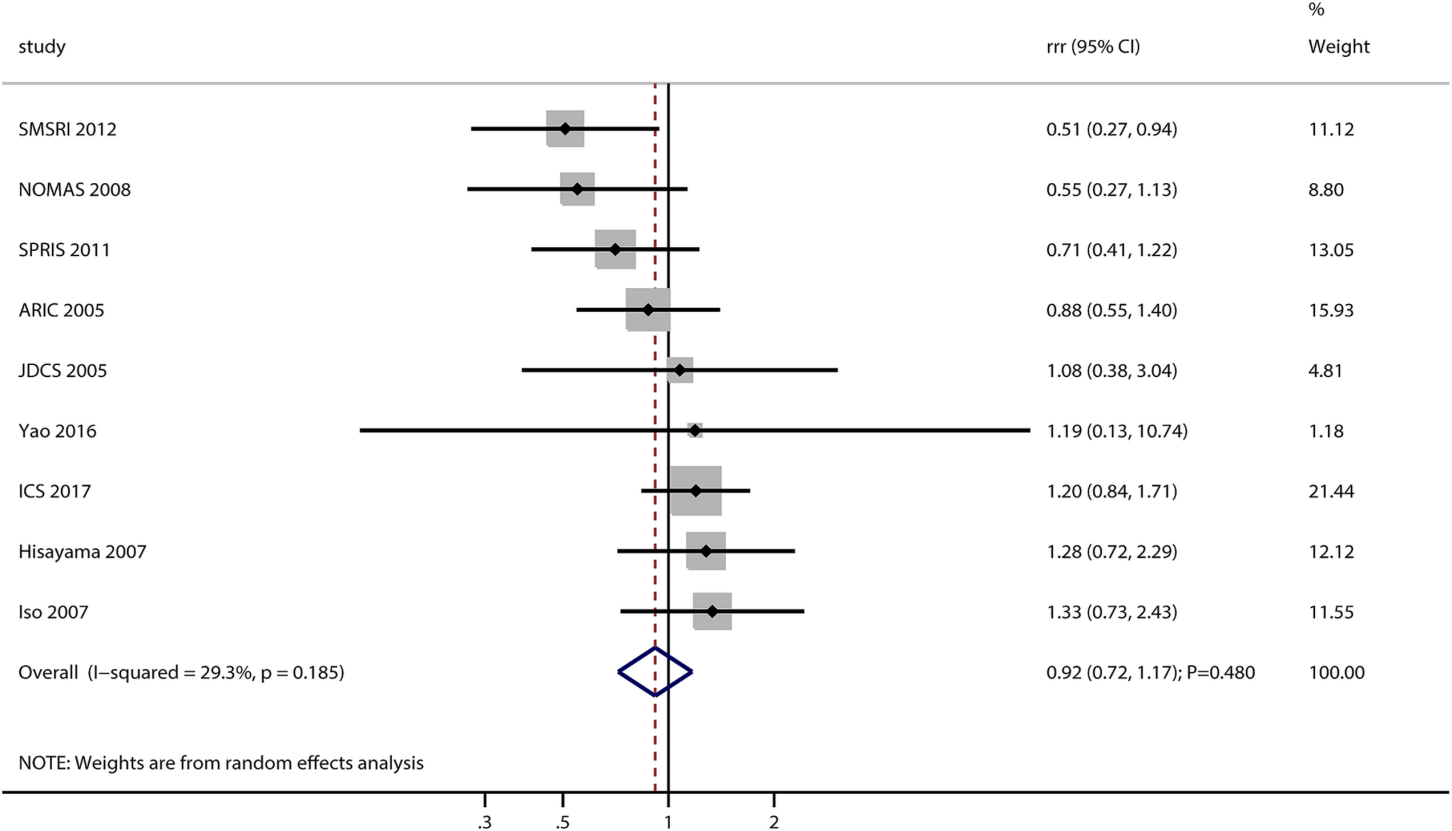
Sex difference in the association between MetS and the risk of stroke.

After pooling all included studies, the sex difference in the association between MetS and the risk of stroke was not significant (RRR: 0.92; 95% CI: 0.72–1.17; *p* = 0.482; Figure 2). No significant heterogeneity was observed across the included studies (*I^2^* = 29.3%; *p* = 0.185). Sensitivity analysis found that the pooled conclusion was stable and not altered by sequential removing a single study (data not shown).

### Subgroup analysis

Subgroup analysis for the sex difference in the relationship between MetS and the risk of stroke is shown in Table 2. No significant sex difference was observed in the association between MetS and the risk of stroke in most subgroups. However, sex difference was found when the follow-up duration was <10.0 years (RRR: 0.70; 95% CI: 0.46–1.04; *p* = 0.078) and in studies with low quality (RRR: 0.54; 95% CI: 0.30–0.98; *p* = 0.043).

**Table 2:** Subgroup analyses for diagnostic performance.

Factors	Groups	RRR	*p* value	Heterogeneity (%)	*p* value
Country	Eastern	0.98 (0.74–1.31)	0.892	31.4	0.188
	Western	0.75 (0.49–1.15)	0.194	10.5	0.291
Age (years)	≥ 55.0	0.94 (0.61–1.47)	0.792	9.3	0.346
	< 55.0	0.90 (0.65–1.24)	0.516	50.0	0.092
Smoking (%)	≥ 25.0	0.95 (0.67–1.35)	0.768	37.0	0.175
	< 25.0	0.86 (0.57–1.30)	0.472	39.6	0.175
Adjusted level	High	0.82 (0.55–1.22)	0.321	53.1	0.074
	Low-moderate	1.07 (0.82–1.41)	0.624	0.0	0.781
Outcomes	Total stroke	0.83 (0.63–1.09)	0.185	5.5	0.381
	Ischemic stroke	0.96 (0.71–1.30)	0.803	29.8	0.223
Follow-up duration (years)	≥ 10.0	1.01 (0.76–1.34)	0.949	32.7	0.191
	< 10.0	0.70 (0.46–1.04)	0.078	0.0	0.579
Study quality	High	0.99 (0.79–1.25)	0.959	14.8	0.317
	Low	0.54 (0.30–0.98)	0.043	0.0	0.465

### Publication bias

**Figure 3:**
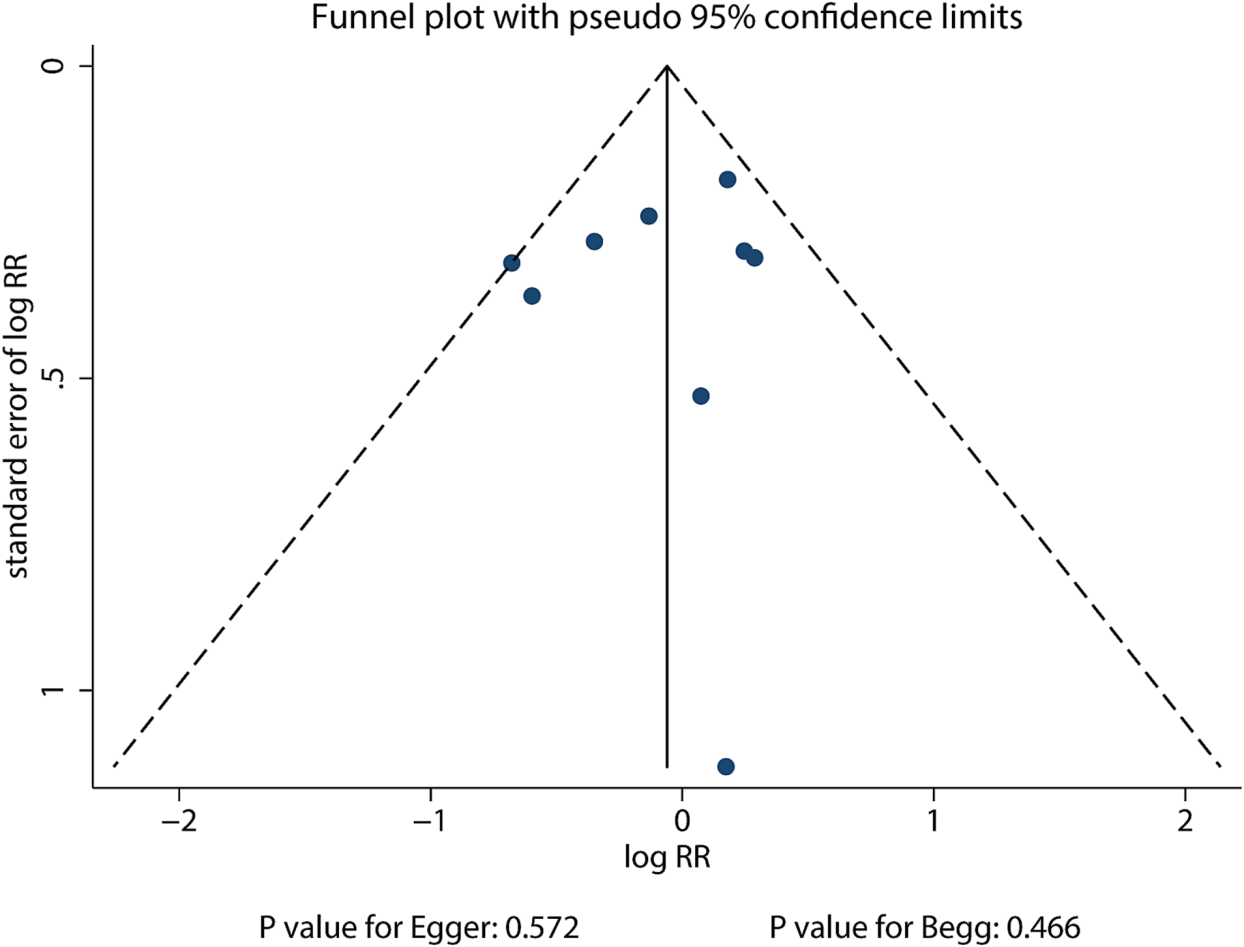
Funnel plot for the sex difference in the association between MetS and the risk of stroke.

The funnel plot for the publication bias of sex difference in the relationship between MetS and the risk of stroke is shown in Figure 3. No significant publication bias was found for the sex difference in the relationship between MetS and the risk of stroke (*p* value for Egger: 0.572; *p* value for Begg: 0.466).

## Discussion

The current systematic review and network meta-analysis was designed to assess the sex difference in the relationship between MetS and the risk of stroke, and a total of 61,060 individuals and 1,770 cases of stroke from 9 studies were identified. This study found no sex difference in the relationship between MetS and the risk of stroke. Sensitivity analysis revealed that the pooled conclusions were stable and not influenced by any single included study. Subgroup analysis found that in subgroups with a follow-up period of <10 years and in subgroups combining low-quality studies, the risk of stroke in men with MetS was lower than that in women with MetS.

The risk of stroke in patients with MetS is significantly increased, as confirmed by previous studies (12). The main mechanisms linking MetS and stroke include the following: (1) Insulin resistance as a core feature of MetS: it leads to high blood glucose and hyperinsulinemia, which in turn promote endothelial dysfunction, inflammation, and the development of atherosclerosis, increasing the risk of stroke (33). (2) Hypertension as a key component of MetS: long-term hypertension damages the vascular walls, leading to vessel hardening and narrowing and increasing the risk of cerebral vascular rupture or blockage (34). (3) Patients with MetS often have high triglyceride levels and low HDL levels; these lipid abnormalities promote atherosclerosis and increase the risk of thrombosis, raising the incidence of stroke (35). (4) Excess abdominal fat associated with chronic low-grade inflammation triggers systemic inflammatory responses, further damaging endothelial function and promoting atherosclerosis (36). (5) Chronic low-grade inflammation and oxidative stress damage endothelial cells, accelerate atherosclerosis, and increase the risk of thrombosis (37). (6) Patients with MetS often have elevated fibrinogen levels and impaired fibrinolytic system function, increasing the risk of thrombosis and, consequently, the risk of stroke (38). Finally, (7) high homocysteine levels and renal dysfunction, among others, can also increase the risk of stroke through different mechanisms (39, 40).

In the previous meta-analysis, subgroup analysis found that male patients with MetS had a significantly lower risk of stroke than female patients with MetS (12). However, our study found that although male patients with MetS had a lower risk of stroke than female patients, this difference was not statistically significant. The reasons for this discrepancy may include the following: (1) The results of the previous studies were based on different populations, which may have led to significant selection bias, affecting the accuracy of the findings. (2) In the previous studies, the ARIC study population was included multiple times, which may have overestimated the sex differences in the association (24).

In the subgroup analysis, we found that in studies with shorter follow-up periods, the association between MetS and stroke may differ by sex. The main mechanisms for this difference are as follows: (1) the short-term protective effect of estrogen in pre-menopausal women may be more pronounced. Estrogen can regulate vascular function, reduce inflammation, and inhibit oxidative stress, which may contribute to the lower stroke risk in women during this period. As the follow-up time extends, other factors may gradually override the impact of estrogen, leading to a less obvious sex difference (13, 41). (2) Women typically have smaller blood vessels than men, and these vessels may be more susceptible to damage in the short term. This difference may make women more vulnerable to stroke in the short term (42). Finally, (3) women may be more susceptible to acute stress and pressure in certain situations, which can increase the risk of cardiovascular events, including stroke, in the short term (14). In the low-quality study subgroup, the limited number of studies (only two studies) may have led to more variable results. The small sample size in these studies may not be sufficient to accurately represent the true relationship between MetS and stroke risk in different genders, resulting in the observed sex differences. Further research with a larger number of high-quality studies is needed to clarify this association.

In addition to the factors already considered, several other variables can influence the association between MetS and stroke risk. Socioeconomic factors play a crucial role. Lower socioeconomic status is often associated with limited access to quality healthcare, higher levels of stress, and a higher prevalence of unhealthy lifestyle habits (43). Men in lower-income groups may be more likely to engage in heavy smoking and excessive alcohol consumption as a coping mechanism for financial stress. In contrast, women in the same situation may experience chronic stress-induced hormonal imbalances, which can contribute to MetS and increase the risk of stroke (44). Lifestyle factors such as physical activity and diet also vary between genders and can impact this association. Physical inactivity is a major risk factor for MetS (45). However, males and females may face different barriers to regular physical activity. Men may be more likely to engage in high-intensity activities but may also be more likely to drop out due to work-related constraints. Women, on the other hand, may have more family-related responsibilities that limit their time for exercise (46). In terms of diet, men may have a higher intake of processed foods and saturated fats, while women may have a lower intake of essential nutrients like omega-3 fatty acids, both of which can contribute to the development of MetS and subsequent stroke risk (47). Medication use is another important consideration. Medications used to treat components of MetS, such as antihypertensive drugs and lipid-lowering medications, may have different effects in males and females (48, 49). Based on these differences, sex-specific interventions may be more effective. For women, stress-management programs could be beneficial in reducing stress-related cardiovascular risks. Since women may be more affected by stress-induced hormonal changes, these interventions can help mitigate the impact on MetS and stroke risk (50). For men, interventions focused on smoking cessation and reducing alcohol consumption, may have a greater impact (51). By targeting these specific lifestyle factors, we can potentially develop more effective strategies for preventing stroke in both men and women with MetS.

The potential sex differences in the association between MetS and stroke risk have important implications for clinical practice. Healthcare providers should be aware of these differences when evaluating and managing patients with MetS. For pre-menopausal women, interventions to maintain overall health, such as regular exercise, a balanced diet, and stress management, may help preserve the protective effects of estrogen on the cardiovascular system. For men, especially those with MetS, more intensive management of risk factors such as smoking cessation, controlling alcohol intake, and strict blood pressure control should be emphasized. Tailored health education programs can be developed to target men and women with MetS, providing them with gender-specific information about stroke prevention and lifestyle modifications. This approach can potentially improve the effectiveness of stroke prevention strategies and ultimately reduce the burden of stroke in the population.

This study had several limitations. First, the study outcomes included total stroke and ischemic stroke. Given that the mechanisms of different types of stroke vary, this can significantly influence the sex differences in the risk of stroke associated with MetS. Second, the severity of MetS varies among patients, and the characteristics of the populations studied differ. These variations can influence the risk of stroke. Fourth, this study was not registered, and its transparency was restricted. Finally, meta-analyses based on published articles have inherent limitations, including restricted detailed analyses and inevitable publication bias.

## Conclusion

This study found that the sex difference in the association between MetS and the risk of stroke might have existed when the follow-up duration was <10.0 years and in low-quality studies. However, due to the limitations of this study, further research is warranted. For a more in-depth understanding of the sex differences in the association between MetS and stroke risk, future research should focus on several key areas. Hormonal mechanisms need further exploration. Future studies could delve into the detailed molecular pathways by which estrogen affects the cardiovascular system in pre-menopausal women and how the post-menopause decline in estrogen increases stroke risk. This could involve studying the interaction between estrogen and signaling pathways related to atherosclerosis, inflammation, and endothelial function. Furthermore, prospective studies with longer follow-up periods are essential. They can better clarify the long-term impact of MetS on stroke risk in different genders and determine whether the sex differences observed in short-term studies persist over time.

## Data Availability

The original contributions presented in the study are included in the article/supplementary material, further inquiries can be directed to the corresponding author.
